# Process Design of a Ball Joint, Considering Caulking and Pull-Out Strength

**DOI:** 10.1155/2014/971679

**Published:** 2014-07-03

**Authors:** Bong-Su Sin, Kwon-Hee Lee

**Affiliations:** Department of Mechanical Engineering, Dong-A University, Busan 604-714, Republic of Korea

## Abstract

A ball joint for an automobile steering system is a pivot component which is connected to knuckle and lower control arm. The manufacturing process for its caulking comprises spinning and deforming. In this study, the process was simulated by flexible multibody dynamics. The caulking was evaluated qualitatively through numerical analysis and inspecting a plastically deformed shape. The structural responses of a ball joint, namely, pull-out strength and stiffness, are commonly investigated in the development process. Thus, following the caulking analysis, the structural responses were considered. In addition, three design variables related to the manufacturing process were defined, and the effects of design variables with respect to pull-out strength, caulking depth, and maximum stress were obtained by introducing the DOE using an *L_9_* orthogonal array. Finally, the optimum design maximizing the pull-out strength was suggested. For the final design, the caulking quality and the pull-out strength were investigated by making six samples and their tests.

## 1. Introduction 

An automobile ball joint is a pivoting element used to allow rotational motion between the parts of the steering and suspension systems. Depending on the car development, in general, most automobile ball joints are made of a socket, bearing, plug, and ball stud. The manufacturing process assembling these parts is called the caulking process.

Existing studies [1–4] have tried to simulate the caulking process, because it can affect the performance of the ball joint. Based on the simulation results, it is possible to evaluate the caulking process, qualitatively, by inspecting the plastically deformed shape. The numerical analysis of a ball joint is a set of coupled problems of rigid body and flexible body. In this research, 3D flexible multibody dynamic analysis for a caulking process using cold forging was simulated by DAFUL [5], which adopts an implicit integration method. In general, it is known that results calculated from an implicit solver have less noise and are more stable [5–7].

The structural responses of stiffness and pull-out strength were then calculated, to check if the design satisfies the related requirements. The analysis was sequentially performed, following the caulking process. In this process, the deformation and stress results obtained from the analysis were saved. Sequential analysis has a strong advantage, in that it can be analyzed by considering the deformed shape and residual stress. The axial and lateral forces were applied to obtain its stiffness. This axial stiffness and this lateral stiffness can be measured by the axial displacement and lateral displacement, respectively, which are generated in the ball stud when the specified loads are applied. The pull-out strength means the required force to pull the ball stud out from the ball joint assembly. A low pull-out strength can deteriorate the structural stability and safety performances [2, 3].

Three process variables in caulking were set up as design variables. These were the revolution speed, downward distance, and downward time of the roller. The process variables in the existing products were determined through empirical case study, not by the systematic design methodology proposed in the research. That is, the process variables in the existing products were set up by changing them 5 to 10 times, performing dynamic analyses, and selecting one adequate case. The effects of design variables with respect to pull-out strength, deformed depth of socket, and maximum stress generated in the socket were obtained by introducing the DOE. The DOE is a statistical technique used to study the effects of multiple variables simultaneously [8–10]. In this research, the extent of the DOE is confined to conducting orthogonal array experiments and the ANOVA and selecting optimum levels. Furthermore, conducting experiment in this study means performing numerical simulation. In this research, *L*_9_ orthogonal array was utilized, in which it is assumed that there is no interaction between design variables.

Then, nine flexible multibody dynamic analyses using DAFUL were performed. Based on the simulation results, the design sensitivity over the design range was determined. In addition, the optimum process variables were suggested. Because of such design characteristics and the calculation time of flexible multibody dynamics, no numerical optimization algorithm could be applied. The process variables in this study were suggested by applying DOE scheme, which could replace 27 dynamic analyses with 9 analyses thus reducing the number of analyses by one-third while achieving the similar result. This research focuses on the improvement of structural responses, considering the caulking process and guaranteeing the caulking quality.

## 2. Initial Design and Analysis of the Ball Joint 

### 2.1. Initial Design of the Ball Joint

**Figure 1:**
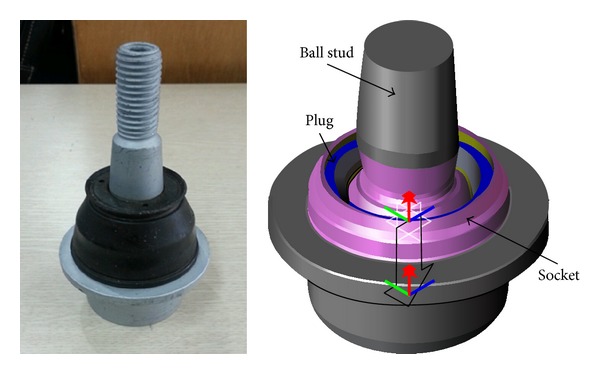
Initial design and components of the ball joint.

The ball joint of the present study is the part mounted on a pickup truck being produced at *A* company. The ball joint investigated in this research is a part that is connected to a knuckle and a lower control arm. The ball joint serves as a flexible pivot element for the steering system. The initial design of the ball joint is shown in Figure 1. The ball joint is made of a socket, bearing, plug, and ball stud as shown in Figure 1.

A plug prevents the components from being separated during manufacture and operation of the ball joint. The bearing, which has relatively much less stiffness among the components of the ball joint, acts as lubrication and buffering. The socket serves as the body of the temporarily assembled ball joint and plays a role in covering the interior parts with the plug through plastic deformation. The ball stud, which induces rotation in all directions, is made by assembling the upper ball and bearing. Each part and the assembly shape of the ball joint are represented in Figure 1 [1–3].

### 2.2. Equations for Dynamic Analysis [2, 6, 7]

The virtual work, *δw*, done by a generalized force is represented as

(1) $$\begin{array}{c} \delta w=\delta u\cdot P^{*}, \end{array}$$

where *δ* **u** is the virtual displacement and P* is the generalized force. The Lagrange multiplier method yields the governing equation for dynamic analysis, called the equations of motion, as follows:

(2) $$\begin{array}{c} F=M\overset{..}{u}+Ku-P+\Phi_{u}^{T}\lambda =0, \end{array}$$

where **M** is the mass matrix, **K** is the stiffness matrix, Φ is the constraint equation, and ***λ*** is a Lagrange multiplier. The positive level constraint equations are represented as

(3) $$\begin{array}{c} \Phi \left(u_{n},\alpha_{n},t_{n}\right)=0. \end{array}$$

Then, the following equations for each step are defined by applying the tangent space method to (2) and (3):

(4) $$\begin{array}{c} H\left(x_{n}\right)=\left[\begin{array}{c} F\left(u_{n},v_{n},a_{n},\alpha_{n},\lambda_{n},t_{n}\right)\\ \Phi \left(u_{n},\alpha_{n},t_{n}\right)\\ \dot{\Phi }\left(u_{n},v_{n},\alpha_{n},{\dot{\alpha }}_{n},\;t_{n}\right)\\ \overset{..}{\Phi }\left(u_{n},v_{n},a_{n},\alpha_{n},{\dot{\alpha }}_{n},{\ddot{\alpha }}_{n},t_{n}\right)\\ U^{T}\left(u_{n}+\beta_{0}v_{n}+\beta_{1}\right)\\ U^{T}\left(u_{n}+\beta_{0}a_{n}+\beta_{2}\right)\\ U^{T}\left(u_{n}+\beta_{0}{\dot{\alpha }}_{n},+\beta_{1}\right)\\ U^{T}\left(u_{n}+\beta_{0}{\ddot{\alpha }}_{n}+\beta_{2}\right) \end{array}\right]=0. \end{array}$$

To solve (4), Newton-Raphson method is applied. Thus,

(5) $$\begin{array}{c} H_{x}\Delta x=-H. \end{array}$$

The solution is updated as

(6) $$\begin{array}{c} x_{n}^{i+1}=x_{n}^{i}+\Delta x. \end{array}$$

### 2.3. Flexible Multibody Modeling of the Ball Joint

**Figure 2:**
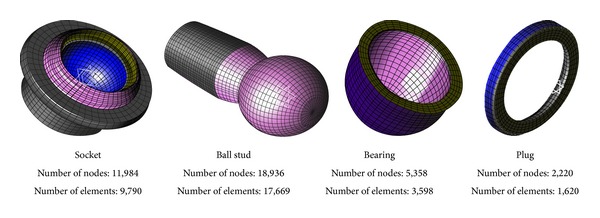
Dynamic model of the ball joint.

**Figure 3:**
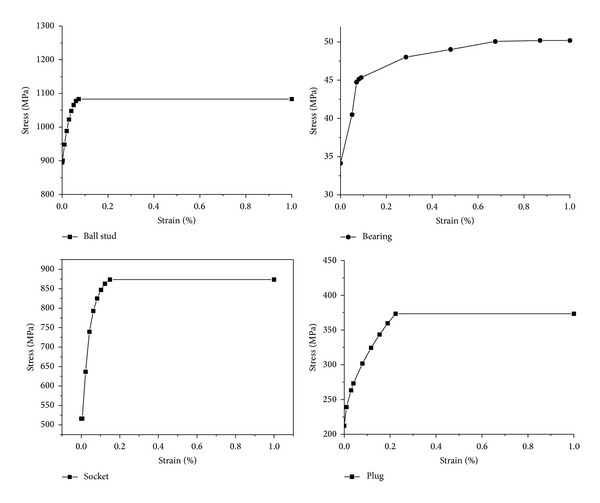
Flow stress-strain curve of each part.

The parts of the ball joint were modeled with hexahedral elements as shown in Figure 2, while two rollers were modeled as rigid bodies. The number of elements of the socket, bearing, plug, and ball stud is 9,790, 3,598, 1,620, and 17,669, respectively. The materials of the socket, bearing, plug, and ball stud are SM45C, nylon, SPC1, and SCM435 [1–3], respectively. The stress-strain curve of each material is represented in Figure 3.

For the contact of the ball joint, there are the contact surfaces between 4 parts, composed of the bearing-ball stud, socket bearing, socket plug, and bearing plug. Each contact surface is defined as a 3-dimensional side. These contacts are defined as “Flex to Flex” to define the contact condition between flexible bodies in DAFUL. In contrast, the contact between roller and socket is defined as “Flex to Rigid” to consider the contact surface between the roller, modeled as a rigid body, and the socket modeled as a flexible body.

### 2.4. Simulation for the Caulking Process and Prediction of the Pull-Out Strength and Stiffness

**Figure 4:**
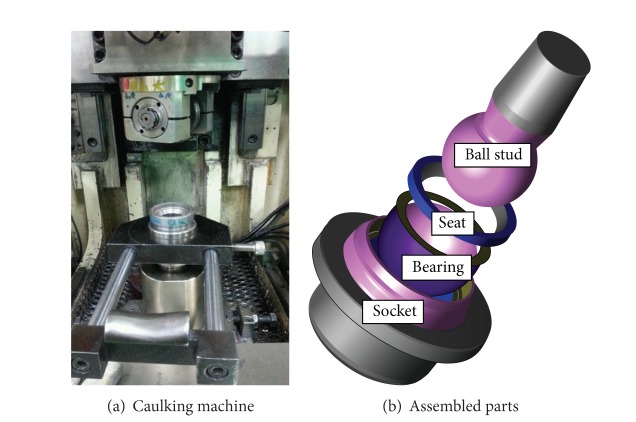
Caulking machine and assembled parts.

**Figure 5:**
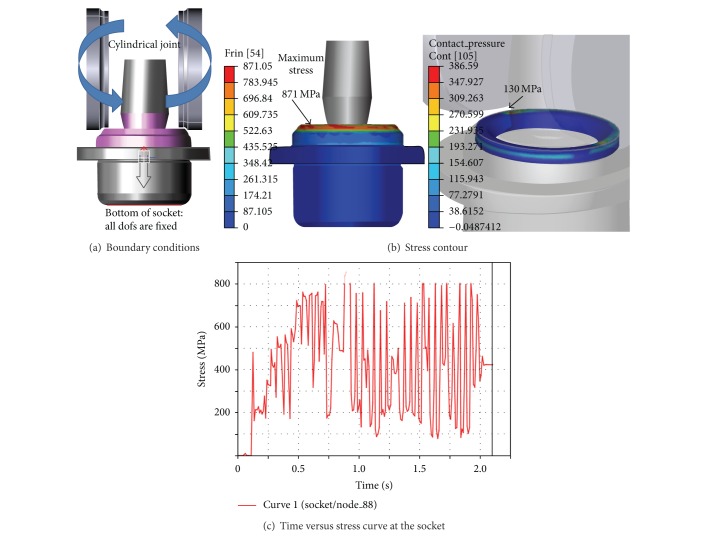
Caulking analysis results.

The manufacturing process of a ball joint is called the caulking process. A caulking machine, as shown in Figure 4, is used to assemble the parts of a ball joint. The parts of the socket, bearing, plug, and ball stud are sequentially positioned in the caulking machine. The lower part of the socket is fixed to a jig. Then, two rollers push down in a vertical direction, to compress the assembled ball joint and plastically deform the upper part of the socket. The roller is set to 300 rpm. The caulking process was simulated by using DAFUL. The movement of each roller is constrained to a CJ (cylindrical joint). The boundary conditions for caulking analysis are represented in Figure 5(a).

The caulking analysis of the initial design requires 38 hours of run-time on a 3 GHz PC. The maximum stress in the ball joint was 871 MPa, which was generated at the surface of the socket. The value was determined from the plastic deformation region. In contrast, the maximum stress at the plug was 130 MPa. Both stresses are shown in Figure 5(b). The stress variation due to time at the socket is represented in Figure 5(c). The ball stud should be securely and stably held in the socket after the caulking process. The performance can be evaluated qualitatively through inspection. Based on the deformation results, it was seen that the caulking of the initial design was successful.

**Figure 6:**
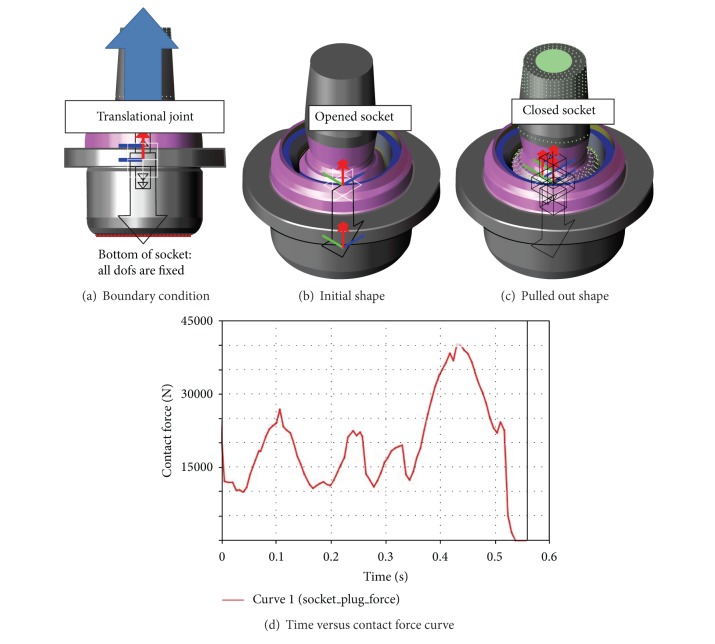
Pull-out strength analysis.

The force to pull out the ball stud of the assembly must be greater than a specified value in order to maintain car performance. The specified value is determined according to the design requirement set by the manufacturer. If the force required to pull out the ball stud is below the specified value, it is considered as a risk that the ball joint could fail during driving. That would make the car lose suspension performance of a lower control arm. The boundary condition for the pull-out strength analysis, initial shape, and pulled out shape is shown as in Figures 6(a), 6(b), and 6(c), respectively.

The specified displacement was given to the center of the ball joint as the loading condition. In this analysis, the contact force between the ball stud and plug was defined as the pull-out force when the ball stud started to be pulled out. The maximum contact force was calculated as 39 kN. The variation of contact force according to time is represented in Figure 6(d), which satisfies the requirement.

**Figure 7:**
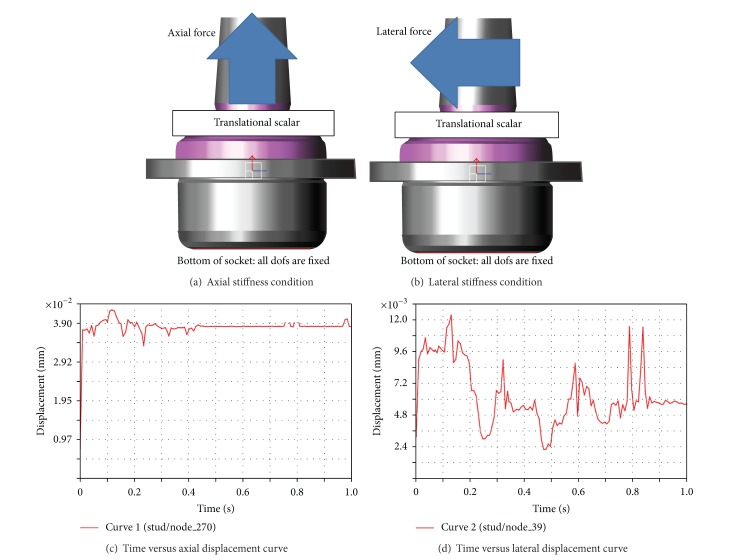
Stiffness analysis condition and results.

Axial and lateral forces are applied to the ball stud, to determine the stiffness of the ball joint. The two force conditions are represented in Figures 7(a) and 7(b). The displacement variations are shown in Figures 7(c) and 7(d). The maximum displacement of each case is much less than its criterion, which is very marginal with respect to the allowable value, therefore satisfying the design requirements for both types of stiffness. Thus, the stiffness performance was excluded from the design constraint, when the DOE was applied.

## 3. Application of Design of Experiments to CAE Based Design 

### 3.1. Definition of Process Variables and Determination of Responses

The roller speed, *A*, the downward distance of the roller, *B*, and the downward time, *C*, were set up as the process variables to find an optimal condition. The downward distance of the roller means the length between the lowest point of the roller and the highest point of the socket. Thus, the process variables, *B* and *C*, determine the descending velocity of the roller.

The most interesting response was the pull-out strength, which was measured by force unit. In addition, the caulking depth and the maximum stress were considered as characteristics. The caulking depth is the bent length of the socket. The longer it is, the better the quality of the caulking becomes. In contrast, the maximum stress is the stress generated in the plastically deformed socket.

### 3.2. DOE Using Orthogonal Array

The overall design process to find an optimum condition is as follows. First, the smallest orthogonal array was selected as the orthogonal array with minimum experiments that can assign all the design variables to their columns. For each process variable, the number of levels was set to three. The second level was set up as the initial variable. The first and third levels were fixed by the lower and upper ones, respectively, around the initial value [8].

The levels of design variables for an orthogonal array were determined as shown in Table 1. Then, an appropriate orthogonal array was selected. For a problem with three variables and three levels, the *L*_9_(3^4^) orthogonal array is recommended, in which two variables are assigned for the first two columns and the remaining variable for the fourth column. The *L*_9_(3^4^) orthogonal array can replace 3^3^ full-combinational experiments.

Second, 9 dynamic analyses were performed as indicated in Table 2. An experiment in this study means one dynamic analysis using DAFUL [6]. It is assumed that the interaction caused by variables can be ignored. That is because just one caulking analysis requires computation time of between 35 and 45 hours, depending on the contact conditions. Almost 15 days were required to finish 9 analyses using DAFUL, including the caulking and pull-out analyses. Each response was obtained as in Table 2.

**Figure 8:**
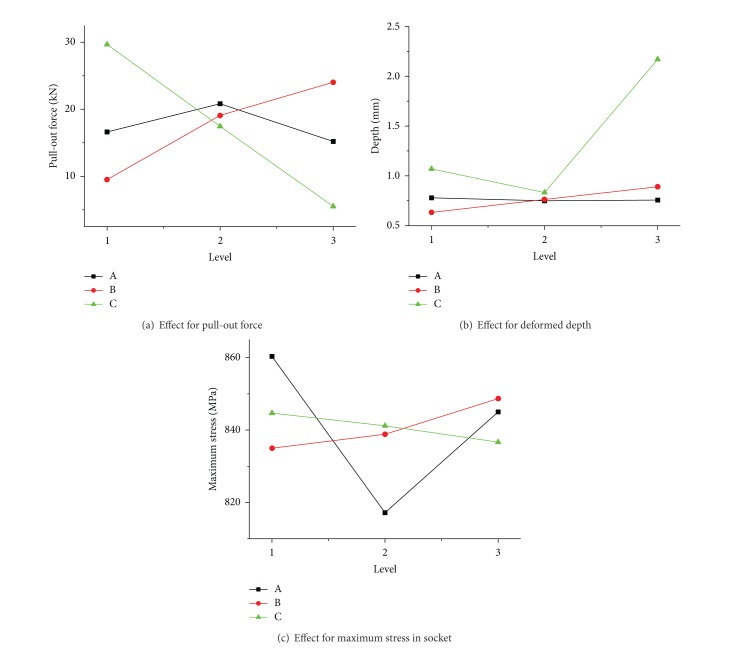
Effect of each variable.

Third, based on the analyses results, the relative importance of each variable to each response could be obtained through an ANOVA. The ANOVA tables for pull-out strength, caulking depth, and maximum stress are shown as Tables 3, 4, 5, 6, 7, and 8, in which significance was established at the 95% confidence level. For the pull-out strength, it is seen from Tables 3 and 4 that the most sensitive variable is *C*, and the variable *A* is insignificant. Therefore, for the caulking depth performance, the variable *C* is considered as the major influence. Considering the maximum stress, only the variable *A* was determined as the significant one. However, the variation of the stress values over the interested range is not large, in comparison with the other responses. Thus, the response of the maximum stress was not considered, when determining an optimum process. The average characteristics for each level called the factor effects are shown in Figure 8. For example, the average pull-out force for the 1st level of design variable *A* was calculated as (22.5 + 19.3 + 8.1)/3. It means that the design variable with large difference between levels has a large influence on the experiments.

The fourth step is to determine the optimum level of each design variable. For the pull-out force, the optimum level is determined as the level with maximum mean from Table 9. Considering only the pull-out strength and the significant variables, an optimum setting is *B*_3_ *C*_1_, since the level with the largest pull-out force can be an optimum level. However, an optimum setting considering only the caulking depth is *B*_3_ *C*_3_. The process variable *B* has the same optimum level for the pull-out strength and the caulking depth. Then, the process variable *C* has an inconsistent optimum level. However, it is seen that the main effect of *C* for the pull-out force is much larger than that for the caulking depth. Thus, an optimum setting of significant variables was selected as *B*_3_ *C*_1_. Also, the variable *A* was selected as *A*_2_. Thus, the optimum levels were determined as *A*_2_ *B*_3_ *C*_1_. Then, the predicted response at the optimum setting was determined as [8–10]

(7) $$\begin{array}{c} f_{\text{pred}}=\mu_{f}+a_{i}+b_{j}+c_{k}, \end{array}$$

where *f*_pred_ is a predicted value of characteristic, *μ*_*f*_ is the overall mean, and *a*_*i*_, *b*_*j*_, and *c*_*k*_ are mean of the main effects of *A*, *B*, and *C* at *i*, *j*, and *k*th level, respectively. The predicted response determined from (7) and the true responses determined from DAFUL are summarized in Table 10. Table 10 shows that the predicted responses at the optimum calculated through DOE are comparable with the true values determined from DAFUL. It can be seen that the difference between the estimated value and the true value is relatively small. Both of them satisfy the requirement specified by the manufacturer.

### 3.3. Test Results for Pull-Out Strength

**Figure 9:**
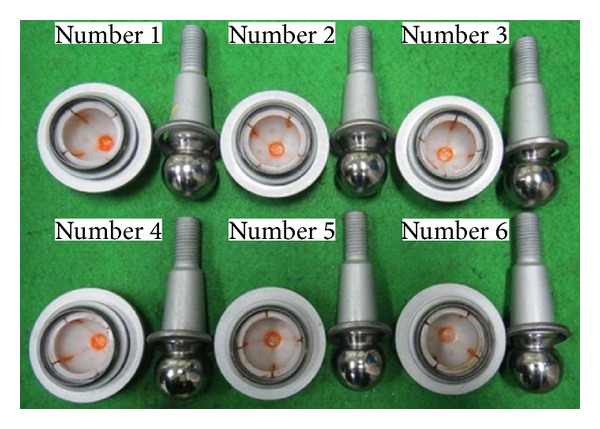
Six specimens for pull-out strength test.

Based on the suggested optimum design, six samples were made to validate the simulation results of the caulking quality and the pull-out strength. From the deformation shapes of the sockets, it was seen that the caulking processes in all the samples were successful. Then, the pull-out strength test was carried out for six specimens. The loading and boundary conditions are the same as the ones of the simulation. The pull-out forces for six specimens are summarized in Table 11. The error between the average value obtained from six tests and the simulation result is about 17%. The specimens after pull-out strength test are shown as in Figure 9.

## 4. Conclusions 

In this study, the caulking process of a ball joint mounted on a pickup truck was simulated by applying the flexible multibody dynamic software called DAFUL. Then, the structural responses of pull-out strength and stiffness were calculated, following the caulking analysis. Sequential analysis has the strong advantage that it can be analyzed by considering the deformed shape and residual stress. Furthermore, three variables in the caulking process were defined as design variables to find their optimum settings. The pull-out strength and the caulking depth were considered in this design stage. Because the flexible multibody dynamic analysis for the caulking process requires a much computation time, an orthogonal array-based design was adopted to economize the computation time. By performing *L*_9_ experimental design, the sensitivity of each process variable to each characteristic was obtained. Then, by the rule of the DOE, the optimum setting of three variables was determined as* A*_2_ *B*_3_ *C*_1_. Based on the suggested optimum design, the caulking quality and the pull-out strength were investigated by making six samples and their tests. The method used in this research can achieve the equivalent result using the existing method requiring two-third less number of analyses.

## Figures and Tables

**Table 1:** Levels of process variables.

Level	*A* (rpm)	*B* (mm)	*C* (s)
1	200	2.7	0.1
2	300	2.9	0.5
3	400	3.1	0.9

**Table 2:** *L*_9_(3^4^) orthogonal array.

	Process variable				Pull-out force (kN)	Caulking depth (mm)	Maximum stress (MPa)
	*A*	*B*	Error	*C*			
1	1	1	1	1	22.5	0.96	866
2	1	2	2	2	19.3	0.87	847
3	1	3	3	3	8.1	0.51	868
4	2	1	2	3	1.9	0.26	795
5	2	2	3	1	31.5	1.04	823
6	2	3	1	2	29.0	0.95	833
7	3	1	3	2	4.1	0.68	843
8	3	2	1	3	6.5	0.38	846
9	3	3	2	1	35.0	1.21	845

**Table 3:** ANOVA table for pull-out strength.

Factor	*S*	dof	*V*	*F*_0_
*A*	51.19	2	25.59	1.26
*B*	326.76	2	163.38	8.07
*C*	874.8 7	2	437.43	21.63
Error	40.44	2	20.22	

**Table 4:** Pooled ANOVA table for pull-out strength.

Factor	*S*	dof	*V*	*F*_0_	*F*(0.05)
*B*	326.76	2	163.38	7.13	6.94
*C*	874.87	2	437.43	19.09	6.94
Error	91.63	4	22.90		

**Table 5:** ANOVA table for caulking depth.

Factor	*S*	dof	*V*	*F*_0_
*A*	0.0015	2	0.0007	0.73
*B*	0.0988	2	0.0494	48.86
*C*	0.7300	2	0.3650	361.00
Error	0.0020	2	0.0010	

**Table 6:** Pooled ANOVA table for caulking depth.

Factor	*S*	dof	*V*	*F*_0_	*F*(0.05)
*B*	0.0988	2	0.0494	56.29	6.94
*C*	0.7300	2	0.3650	415.83	6.94
Error	0.0035	4	0.0009		

**Table 7:** ANOVA table for maximum stress.

Factor	*S*	dof	*V*	*F*_0_
*A*	2868.8	2	1434.42	4.52
*B*	299.1	2	149.58	0.47
*C*	97.3	2	48.66	0.15
Error	633.8	2	316.91	

**Table 8:** Pooled ANOVA table for maximum stress.

Factor	*S*	dof	*V*	*F*_0_	*F*(0.05)
*A*	2868.8	2	1434.42	8.35	5.14
Error	1030.3	6	171.72		

**Table 9:** Mean of pull-out force to each level.

Design variable	Average pull-out force (kN)		
	1	2	3
*A*	16.6	**20.8**	15.2
*B*	9.5	19.1	**24**
*C*	**29.7**	17.5	5.5

**Table 10:** Predicted and true values at optimum variables.

	Pull-out force (kN)	Caulking depth (mm)	Maximum stress (MPa)
Predicted value	39.4	1.19	829
True value	37.1	0.98	833

**Table 11:** Pull-out forces of six specimens.

No. of sample	No. 1	No. 2	No. 3	No. 4	No. 5	No. 6
Pull-out force (kN)	45.5	45.3	45.6	45.6	43.1	43.7
